# Information coding in vasopressin neurons—The role of asynchronous bistable burst firing

**DOI:** 10.1016/j.biosystems.2013.03.010

**Published:** 2013-05

**Authors:** D.J. MacGregor, T.F. Clayton, G. Leng

**Affiliations:** Centre for Integrative Physiology, University of Edinburgh, UK

**Keywords:** Vasopressin, Phasic firing, Dendritic release, Modelling

## Abstract

The task of the vasopressin system is homeostasis, a type of process which is fundamental to the brain's regulation of the body, exists in many different systems, and is vital to health and survival. Many illnesses are related to the dysfunction of homeostatic systems, including high blood pressure, obesity and diabetes. Beyond the vasopressin system's own importance, in regulating osmotic pressure, it presents an accessible model where we can learn how the features of homeostatic systems generally relate to their function, and potentially develop treatments. The vasopressin system is an important model system in neuroscience because it presents an accessible system in which to investigate the function and importance of, for example, dendritic release and burst firing, both of which are found in many systems of the brain. We have only recently begun to understand the contribution of dendritic release to neuronal function and information processing. Burst firing has most commonly been associated with rhythm generation; in this system it clearly plays a different role, still to be understood fully.

## 1 Introduction

**Fig. 1:**
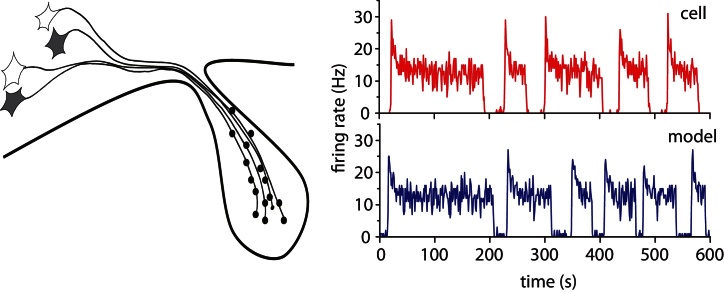
Vasopressin cells project to the posterior pituitary. In response to osmotic input, cells fire in a distinct phasic bursting pattern. We can closely match this behaviour with a relatively simple single cell model.

We now recognise that rather than just simple integrators and relays of activity, most neurons have complex pattern generating properties. An important question in contemporary neuroscience is how these properties contribute to information processing (Ramirez et al., 2004; Buzsáki and Draguhn, 2004). In particular, many neurons generate “bursting” patterns of electrical activity, arising either through intrinsic mechanisms, or via network interactions. Some of these contribute to generating physiological rhythms (such as the respiratory rhythm, Del Negro et al., 2002), where neurons synchronise across a network to generate an emergent rhythm. In others, single synchronised bursts are essential to the physiological output, such as oxytocin cells driving the periodic milk let-down during suckling (Rossoni et al., 2008). However, some neurons, like the vasopressin cells of the hypothalamus, generate bursting activity individually (Fig. 1), and fire *asynchronously* (Leng et al., 2008), such that the bursting is not reflected in the population output – so what is *this* bursting behaviour for? We know in vasopressin neurons that bursts are efficient for stimulus-secretion coupling, optimising secretion per spike, but it is not clear why this is important – many neurons fire spontaneously at much higher rates than vasopressin cells. Moreover, bursting in these cells is efficient for triggering secretion only because of particular properties of their axon terminals, indicating that these secretion properties have co-evolved with the bursting behaviour; suggesting that bursting is important for other reasons. Because bursting is such a widespread feature in the CNS, arising in many different ways, we believe it is important to understand exactly what advantages it offers for information processing.

Vasopressin and its control of osmotic pressure is a relatively simple and very well studied system. It presents an unusually strong opportunity to be able to relate information processing properties of cells to their physiological function as part of a system. We are currently attempting to apply a modelling and complex systems approach, in order to test specific hypothesis about the adaptive value of its particular features (heterogeneity, bistability, autocrine and paracrine communication mechanisms). We will test these features ultimately by expressing the physiological function of the system in terms of a defined control task, integrating neuronal modelling into a physiological systems model. The project is defined in three parts:

1. Build a single neuron model, including spike firing, vasopressin secretion and intercellular communication mechanisms.
2. Duplicate the model to build a network. Evaluate input/output characteristics and study the effects of varied assumptions about communication.
3. Build a closed-loop system model and use this to test varied network models, comparing their performance in matching experimental data, and systematically evaluate network performance, robustness and efficiency.

The first details of these components are presented elsewhere (Clayton et al., 2010). What we seek to develop here is the rationale and strategy behind the work. Though these parts require initially to be developed in sequence, they will continue to be refined in parallel, better informed by their role and behaviour as part of a system. This is an extended version of a paper previously presented at the 9th International Conference on Information Processing in Cells and Tissues (MacGregor et al., 2012).

## 2 Background: homeostatic role of vasopressin

Vasopressin is made by neurons of the supraoptic and paraventricular nuclei of the hypothalamus, and is secreted into the blood from axonal terminals at the posterior pituitary. This is a very important “model system” in neuroscience for many reasons, including the large size and accessibility of the neurons, and the fact that, because these neurons secrete their products at measurable amounts into the systemic circulation, their electrical activity can be directly related to secretion and physiological function. These cells use cell volume modulated stretch-sensitive channels to respond to osmotic pressure, and also receive synaptic input from other osmosensitive neurons (Bourque, 2008).

Vasopressin cells display relatively long bursts and silences. They are bistable oscillators; small perturbations can “flip” a neuron from either state (burst or silence) to the other, because their intrinsic activity-dependent conductances can either stop or start a burst. An asynchronously firing population of vasopressin cells has a lot of potential for interesting signal processing properties. They may act as a low-pass filter – preserving low frequency signals while filtering out stochastic noise in their inputs (Sabatier and Leng, 2007). While individual neurons respond erratically to acute changes, the asynchronicity means that these erratic responses are smoothed out. The vasopressin cells are also a heterogeneous population; variation in expression of membrane channels, receptors, and synaptic input, produce differing sensitivities to osmotic pressure and a wide spectrum of bursting behaviour. This heterogeneity has been preserved through evolution, suggesting either that it is an inescapable limitation, or, more interestingly, that the heterogeneity is adaptive and has some functional purpose. There are some clear functional consequences of heterogeneity – a population that is heterogeneous in osmotic responsiveness will have a wider dynamic range than a homogeneous population. But there are also costs; for example, a homogeneous population has a high intrinsic redundancy, so it is robust to degradation. A heterogeneous population will generally be less robust – unless the heterogeneity is not hard-wired, but arises from network self organisation. The most obvious way that heterogeneity could be self-organised would be if individual neurons cycle through phases of varying osmotic responsiveness – as we have suggested that they might (Leng et al., 2008). The population of vasopressin neurons acts together as a complex system, with multiple feedbacks acting at different levels, including autocrine signals and paracrine signalling between cells. These properties co-ordinate the vasopressin cells, presumably to optimise emergent features of system behaviour.

**Fig. 2:**
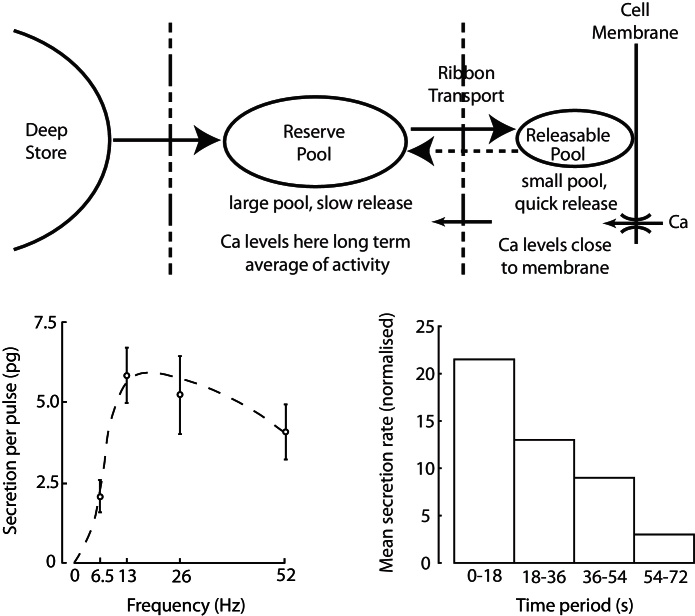
Vasopressin is synthesised in the cell body and transported to the release sites through a sequence of pools, with transport and release activity driven by spike triggered Ca^2+^ entry, and also possibly internal stores. The lower panels show *in vitro* data from Bicknell (1988). Stimulus-secretion coupling is non-linearly dependent on both spike rate (lower left) and burst duration (lower right). Per spike secretion is optimal at ~13 Hz, initially showing facilitation, before being limited by the releasable pool. The secretion rate also declines during prolonged bursts as the reserve pool is depleted.

The vasopressin-osmotic system is part of a larger homeostatic system that regulates plasma volume and electrolyte concentration via many mechanisms (including thirst and natriuresis; see Bie, 2009). Vasopressin secretion (Fig. 2) increases linearly with osmolarity above a set point (Dunn et al., 1973), and this is essential for regulation of plasma volume and osmolarity. Plasma osmolarity is normally regulated to within a few percent, so vasopressin cells, as a population, must respond reliably to a change in extracellular [Na^+^] of just ~1 mM – tiny compared to the fluctuations expected as the result of stochastic variations in neuronal activity. A sustained increase in osmolarity requires a sustained vasopressin response, so the vasopressin cell population must maintain their response to an unvarying input signal. Most neurons are good at responding to change, but to do this they adapt to a constant signal; vasopressin cells as a population must not adapt to sustained osmotic stimulation.

At normal osmotic pressures, the cells fire slowly; each secretes just 1–2 vesicles/s, but this is enough to maintain normal circulating concentration of ~1 pg/ml (see Leng and Ludwig, 2008 for details). As osmotic input increases, the cells enter a bistable phasic firing mode (Sabatier and Leng, 2007), consisting of alternating bursts and silences. Each burst typically lasts for 20–60 s at 4–10 spikes/s. Secretion is facilitated by high frequency spiking, but fatigues within about 20 s; this fatigue is reversed after 20–30 s of quiescence; thus a phasic firing pattern optimises secretion per spike (Bicknell, 1988). A burst of ~400 spikes in one vasopressin cell releases about one vesicle from each of its ~2000 nerve endings. However, with chronic stimulation, the stores of vasopressin are progressively depleted; if rats are given 2% NaCl to drink, then stores decline to ~15% of control values over 12 days, despite a massive increase in synthesis (Kondo et al., 2004). This decline reflects the delay between increasing the rate of synthesis and replenishment of the stores. At any particular time, hormone secretion in response to a given stimulus is proportional to the size of the store (Higuchi et al., 1991); thus, during progressive dehydration, spike activity becomes less and less effective at secreting vasopressin.

The larger homeostatic system, of which the vasopressin system is part, must regulate plasma osmolarity and volume within strict tolerance. Both hyper- and hyponatraemia are life-threatening outside critical limits. We propose that the utility of this system should be judged not by how accurately it maintains normal osmolarity, but by how well it can prolong survival – *i.e.*, when subject to chronic challenge, how long will it maintain osmolarity within tolerable bounds? This is a novel way of understanding the vasopressin system; it expresses its physiological function in terms of a defined control task, and in so doing it enables systematic, objective study of its performance, and systematic assessment of the utility of each of the various features of the vasopressin system.

**Fig. 3:**
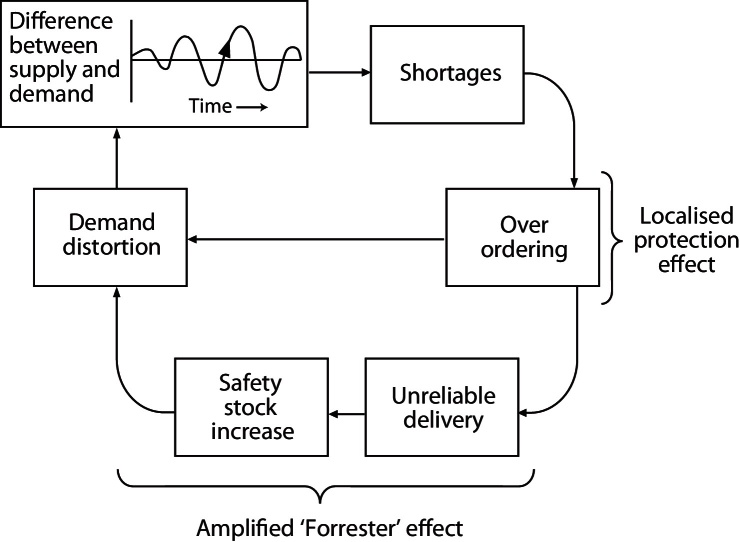
The ‘Forrester flywheel’ summarizes common problems in supply chains (Towill, 1996). In business, stock levels incur space and wastage costs so must be kept low; but if stocks run out, delays in restocking mean lost sales. In response to fluctuations in demand (synaptic input) the business can alter manufacture (synthesis) and moderate supply (secretion) by moderating price levels (stimulus-secretion coupling). Management needs to link manufacturing (synthesis) to sales (secretion); to link stock (stores) levels to a given variability of demand (expected variability of physiological challenge) for given delays in the system; and to ration supply by raising prices. The business must minimise the risks of losses associated with either running out of stock (hypernatraemia) or overstocking.

This task is not trivial, and is in fact analogous to a problem in supply chain management that has considerable economic significance (Fig. 3).

## 3 Network structure and regulation

**Fig. 4:**
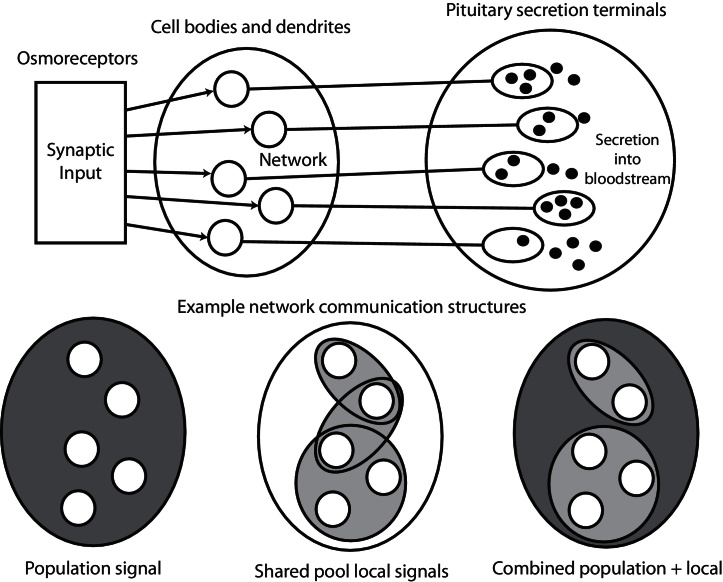
The vasopressin cell population receives synaptic input from osmoreceptor neurons, and intercommunicates via dendritic release. Each cell body has its own release terminal where vesicles are released into the bloodstream. The lower panel shows varied plausible network topologies, making use of dendritic signals; a common population signal, overlapping local signal pools (similar to the oxytocin network of Rossoni et al., 2008), and the two combined.

Vasopressin cells are not synaptically interconnected, but communicate via dendritic release of several substances (Fig. 4). This requires Ca2+ dependent exocytosis of stored vesicles, and during sustained osmotic stimulation this is triggered by spike activity (Ludwig et al., 2002). Vasopressin itself is a paracrine signal; it survives long enough to diffuse to neighbouring neurons and act as a population feedback, exciting slow firing cells and inhibiting those more active (Gouzènes et al., 1998). Dynorphin is packaged in the same vesicles as vasopressin, but in smaller quantities and is broken down more quickly; it is an autocrine regulator, causing a slow inhibition that helps to terminate bursts (Brown and Bourque, 2006). Endocannabinoids (Hirasawa et al., 2004; Di et al., 2005), apelin (Llorens-Cortes and Moos, 2008), galanin (Kozoriz et al., 2006), adenosine (Ruan and Brown, 2009) and nitric oxide (Stern and Zhang, 2005), also modulate spike activity, some by presynaptically inhibiting synaptic input. The mechanisms vary, and differ in duration of effect and spatial dispersion.

We hypothesise that these signals coordinate the heterogeneous activity of vasopressin cells to efficiently encode and respond to osmotic stimuli over a wide dynamic range, over prolonged periods. Heterogeneity in osmosensitivity will in itself extend the dynamic range of the system, but the most active neurons will be depleted relatively rapidly. We suspect therefore that the feedback interactions may ensure that cells, during chronic stimulation, cycle through prolonged stages of activity and rest (as suggested in Leng et al., 2008). Such cycling may arise as an emergent property of network interactions. The network must also compensate for chronic depletion of pituitary stores and be resistant to random degradation (loss of neurons as a result, for example, of aging). We hypothesise that the network interactions will provide robustness to the system as a whole, enabling it to adapt to cell loss.

## 4 The modelling approach

The ‘first focus’ hypothesis for the modelling is that burst firing is an essential element of the neurons’ output response. We believe that bursting is essential for efficient signal encoding but we do not yet understand *why*. The first part of addressing this is to understand the mechanism.

Electrophysiological studies of vasopressin cells (Armstrong, 2007; Bourque, 2008; Tasker et al., 2002) have led to Hodgkin–Huxley type neuron models that closely match *in vitro* data (Komendantov et al., 2007; Roper et al., 2004). Our current single cell model incorporates the basic mechanisms implemented in these, but in a computationally simpler form – a modified leaky integrate and fire model which (with minor variations in parameters) can be tightly fit to *in vivo* data from the whole spectrum of recorded vasopressin cell activities, and which can therefore be duplicated with variation to form a realistic heterogeneous neuron population.

**Fig. 5:**
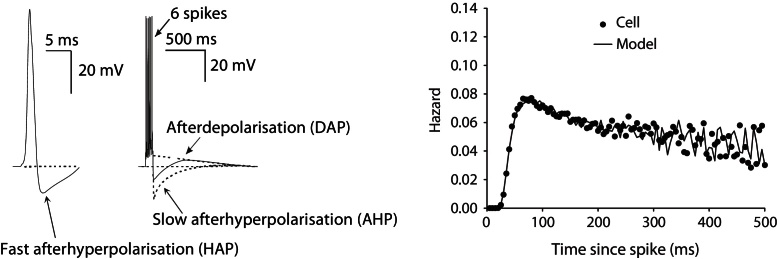
Three major post spike potentials, the HAP, the AHP and the DAP shape the cells’ electrical activity. The hazard, with model fitted to cell, shows how excitability changes post-spike, with shape determined by these post spike potentials (Sabatier et al., 2004). The large magnitude but fast decaying HAP generates the initial refractory period. The DAP generates the following peak in excitability which gradually falls to a plateau.

The simplest leaky integrate and fire model has a single variable and differential equation representing membrane potential; it assumes that excitability only varies with input activity, using a fixed firing threshold, and resetting after each spike. To simulate (mostly calcium driven) post-spike afterpotential dependent changes in excitability observed in vasopressin cells, our modified, non-renewal version, similar to the spike response model of Gerstner (Jolivet et al., 2004), adds three afterpotential variables (Fig. 5), described by ordinary differential equations as decaying exponentials, summed to generate a varied firing threshold. The transient, hyperpolarising afterpotential (HAP) causes a post spike refractory period of ~50 ms. The smaller, slower, after-hyperpolarising potential (AHP) summates to limit firing rate. As the HAP decays, a subsequent depolarising afterpotential (DAP) confers a transient hyperexcitability; this can accumulate across spikes, contributing to the inception and maintenance of bursts. During bursts, the autocrine action of dendritic dynorphin release slowly attenuates the DAP, resulting in a shift in excitability which eventually terminates the burst. The model simulates this by adding a bistable component.

Synaptic input is modelled as a Poissonian mix of excitatory and inhibitory random perturbations to the membrane potential which leaks, decaying back towards resting potential. These perturbations are either fixed amplitude ~1–4 mV or use ionic conductance based reversal potentials. Using these components, the model can match observed activity in varied vasopressin cells, matching (a) spike interval distributions and hazard functions, (b) burst and silence distribution, (c) firing rate index of dispersion, and (d) burst temporal profile. It also matches the functional behaviour of the more complex models, fit to extensive experimental data (Sabatier et al., 2004).

### 4.1 Spiking model equations

The spike model described here is a development of the model presented in Clayton et al. (2010), using a simplified bistable mechanism, and replacing the gaussian noise based input. The membrane potential *V* is the sum of the components described below:

(1) $$V=B+H+A+V_{\mathrm{syn}}$$

A spike is fired when *V* exceeds the threshold parameter *V*_th_. The resting potential is defined as 0, where the model is initialised.

Decaying synaptic input is modelled by:

(2) $$\frac{{\mathrm{dV}}_{\mathrm{syn}}}{\mathrm{dt}}=-\frac{V_{\mathrm{syn}}}{\tau_{\mathrm{syn}}}+e_{n}{\mathrm{syn}}_{\mathrm{mag}}+i_{n}{\mathrm{syn}}_{\mathrm{mag}}$$

where *e*_n_ and *i*_n_ are the Poisson random EPSP and IPSP counts, generated here using the same mean rate, syn_rate_. Parameter syn_mag_ gives the PSP magnitude. Input decays exponentially with half life *λ*_syn_. The time constants are calculated from half life parameters using the formula:

(3) $$\tau_{x}=\frac{\lambda_{x}}{\log_{n}2}$$

where *x* is the variable concerned.

The HAP and AHP variables *H* and *A* are modelled as decaying exponentials with halflife parameters *λ*_*H*_ and *λ*_*A*_, incremented by *k*_*H*_ and *k*_*A*_ when a spike is fired:

(4) $$\frac{d_{H}}{\mathrm{dt}}=-\frac{H}{\tau_{H}}+k_{H}s$$

(5) $$\frac{d_{A}}{\mathrm{dt}}=-\frac{A}{\tau_{A}}+k_{A}s$$

where *s* = 1 if a spike is fired at time *t*, and *s* = 0 otherwise.

A similar equation describes a slow inhibitory variable *I*, representing dynorphin:

(6) $$\frac{d_{I}}{\mathrm{dt}}=-\frac{I}{\tau_{I}}+k_{I}s$$

The DAP uses a similar form, but with its value capped by parameter *D*_cap_, also accounting for the effect of the AHP:

(7) $$\frac{d_{D}}{\mathrm{dt}}=-\frac{D}{\tau_{D}}+\left\{\begin{array}{cc} \frac{D_{\mathrm{cap}}-D-A)}{D_{\mathrm{cap}}}k_{D}s & \mathrm{if}\;D+A<D_{\mathrm{cap}}\\ 0 & \mathrm{otherwise} \end{array}\right)$$

The following equations describe the bistable bursting mechanism:

(8) $$\frac{{\mathrm{dB}}_{\mathrm{syn}}}{\mathrm{dt}}=\frac{\left(V_{\mathrm{syn}}-B_{\mathrm{syn}}\right)}{100}$$

(9) $$B_{i}=B+B_{\mathrm{syn}}$$

(10) $$\frac{\mathrm{dB}}{\mathrm{dt}}=\frac{-(B_{i}-D+B_{1}I)(B_{i}-I)(B_{i}+I)}{100}$$

The bistability variable *B* incorporates the effects of the DAP and opposing dynorphin accumulation, encoding two stable points, bursting and silence, and an unstable balance point. Variable *B*_syn_ adds the random perturbations generated by synaptic input. Parameter values were derived from fitting the model to *in vivo* experimental data using a genetic algorithm, running the model on 1 ms steps (Clayton et al., 2010). An example set, corresponding to Fig. 1, is presented in Table 1.

**Fig. 6:**
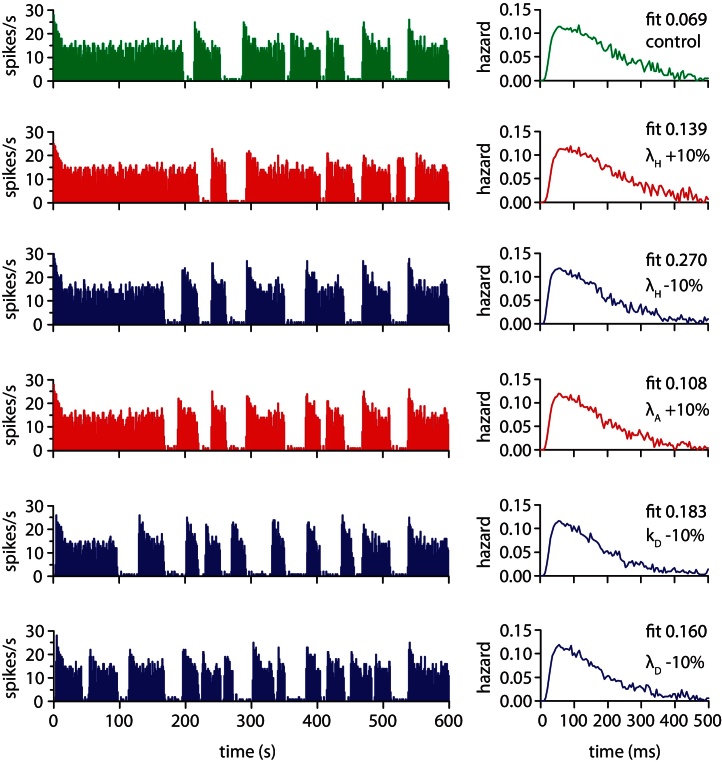
Testing parameter sensitivity (post-spike excitability parameters). We tested each parameter with 10% changes starting with the fit in Table 1. Here we show the spiking pattern and hazard functions for the changes which showed the greatest variation based on the chi square fit measure comparing the model generated hazard function to the *in vivo* data. Each run uses the same randomly generated synaptic input. The control data uses the fitted Table 1 parameters.

**Fig. 7:**
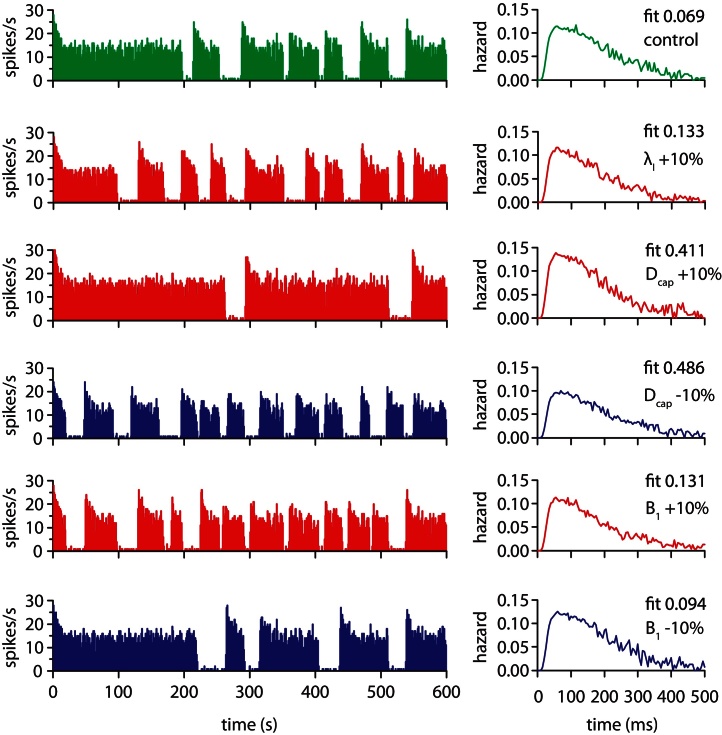
Testing parameter sensitivity (bursting mechanism parameters) as in Fig. 6. Changes in parameter *D*_cap_, which corresponds to the maximal conductance of the slow DAP, show the greatest variation in the hazard function and spiking pattern, with a larger value causing a higher magnitude post-spike depolarisation and resulting longer burst duration.

We tested the robustness of the model and the parameter fit by testing each parameter in turn with 10% changes (Figs. 6 and 7). The results showed some variation in burst duration and short term spike patterning (detected in the hazard function Sabatier et al., 2004) but continued to plausibly represent *in vivo* activity. We assessed how much the activity varied from the fitted control by using a chi square measure to compare the model generated and *in vivo* hazard functions. The figures show the parameter changes which gave the largest variation from the fitted parameter set.

In further work currently under development we are attempting to couple the spiking model to a secretion model based on the physiological mechanisms of vasopressin vesicle trafficking and secretion (Fig. 2). This ordinary differential equation model uses five variables; representing spike dependent changes in [Ca^2+^] concentration, activity dependent facilitation, facilitation clearance, and the size of the releasable and reserve pools. Parameters are derived from experiments, and fitted to match the non-linear stimulus-secretion coupling properties (including facilitation and fatigue) observed *in vitro e.g.* (Bicknell, 1988).

Development and testing of the model is in custom software built in C++ and wxWidgets, based on modelling and data analysis software we have previously developed to study diverse neuroendocrine systems (MacGregor and Leng, 2005; Macgregor and Lincoln, 2008; MacGregor et al., 2009).

### 4.2 The single neuron model and communication mechanisms

In order to simulate a heterogeneous population we must be able to duplicate the single neuron model with variation. The burst firing mechanism must be shown to remain robust under parameter variation, so that we can randomly generate a varied population, introducing variation into the model in a way that closely resembles the heterogeneity observed *in vivo*.

The stimulus-secretion components that have been modelled represent vesicle trafficking and secretion, to reproduce facilitation and fatigue. However, testing the model over longer timescales (hours and days) must also take account of long term depletion of vasopressin stores, and will require modelling of synthesis and transport mechanisms, including long timescale store replenishment by activity-dependent synthesis (Fitzsimmons et al., 1994).

The most novel part of the neuron model will be the network communication mechanisms (dendritic release and response) and tools for building the network's structure. Each released substance will have rules that govern spatial and temporal dissemination, and will have different effects. Most inhibit electrical activity, but by different mechanisms, some by modulating EPSP rate and/or IPSP rate and some post-synaptically (*e.g.* by modulating resting potential or EPSP magnitude). This is the stage where the project becomes more speculative and predictive. We still know very little of the functional purpose of these mechanisms, or their endogenous triggers.

### 4.3 Osmotic signal encoding

The vasopressin neurons receive excitatory synaptic input from osmosensitive neurons and have their own excitatory osmosensitive channels. The neurons also receive inhibitory synaptic inputs, but it is not certain whether these are also osmosensitive. *In vitro*, a hypertonic stimulus applied to the main core of osmosensitive neurons has been shown to increase EPSPs but not IPSPs at the vasopressin neurons (Richard and Bourque, 1995), but combined modelling and *in vivo* study (Leng et al., 2001) suggests that coactivation of excitatory and inhibitory inputs is required to produce the linearity observed in the spike rate response. We can further test these options with the phasic spiking model, including an exploration of whether the intrinsic osmosensitive excitation serves purely as redundancy, or makes some functional difference to osmotic signal encoding.

Finally, the vasopressin neurons interact via dendritic secretion of vasopressin and other factors. We have argued that the behaviour of vasopressin cells is influenced by the activity of their neighbours, through the generation of ‘population signals’. For the encoding, the important output signal is the summed population activity, and communication mechanisms are required to coordinate the response.

### 4.4 Building a robust signal encoding and response network

The simplest population signal is weak mutual interaction, which affects all neurons similarly. Dendritically released vasopressin may be such a signal, given its abundance and long half-life. For paracrine signals (*e.g.* nitric oxide and endocannabinoids), or to test more limited vasopressin dispersion, each neuron will share one or more input pools (Fig. 4), based on dendritic bundles (Ludwig and Leng, 2006). We recently developed a network model to understand how oxytocin cells orchestrate synchronized bursts during reflex milk ejection (Rossoni et al., 2008). In that model, each neuron contacts a few ‘dendritic neighbours’ via one or more shared communication pools (dendritic bundles), with dendritic secretion non-linearly coupled to firing activity; we will use similar network topologies here (Fig. 4).

Choosing to model dendritic communication in this way implicitly assumes some signals are confined within defined dendritic bundles, while other signals are distributed freely throughout the population. The evidence for distinct bundles comes from electron microscopic studies showing that in dehydration, magnocellular neuron dendrites are found directly apposed in bundles of 2–8 dendrites. The bundles are enclosed by astrocyte processes that act to regulate them physiologically. Though we are not proposing to model the dynamics of bundling, this does suggest that some signals may be effectively confined within bundle based pools. We will incorporate this into the model using communication pools as illustrated in Fig. 4.

Neurons contacting two or more pools will link sub-populations, and varying the number of pools, number of neurons in each, and how many each neuron contacts, generates a wide range of structures to test. Randomly connected structures can be built by an automated process based on defined parameters, and cell heterogeneity can be generated by randomly varying, for example, input rates and HAP parameters.

We will test network performance with increasingly difficult tasks, progressively introducing the more complex network structures. An initial network of 100 neurons should be sufficient to test varied structures but small enough to ease analysis, though we will eventually aim to move to larger networks on a comparable scale to the ~9000 neurons *in vivo*. The first objective is a network which responds to a fixed mean input (varied between tests) linearly with a stable maintained response, proportional to the average network input over a wide dynamic range. We have good information on stimulus-secretion coupling in the nerve endings, but know less about stimulus-secretion coupling for the dendrites. However, it is activity dependent (during osmotic stimulation; (Ludwig et al., 2002)), so we initially assume the non-linearity of release is similar to that at the terminals, and that dendritic release is proportional to the size of the stores.

For more advanced tests, input protocols vary the input rate, either in discrete steps or continuously; the network must be able to track these changes without generating unstable feedback cycles (see Forrester flywheel) and maintain a linear output response. Neuronal responses tend to be highly non-linear, and it is still unknown what mechanism might feedback from the secretion response to modulate spike activity. We will test the network further by increasing the variation in the neurons, including excitability, bursting properties, and synthesis and secretion.

### 4.5 Building and testing a vasopressin system

The vasopressin system is very efficient; under most conditions osmotic pressure varies only by 3%, leaving a very small dynamic range for the input signal. It can lose many of its neurons and still maintain response and can also sustain response for several days of prolonged osmotic challenge, despite limited stores. We will formulate the homeostatic control task fulfilled by the vasopressin system in a way that allows us to objectively assess its performance (and compare it with that of related networks), and to study systematically the robustness of the network in the face of (a) increasing levels of noise in the inputs and (b) progressive degradation (cell death as occurs naturally during aging), modelled as either random or activity-dependent (excitotoxic) neuronal loss.

There have been many previous attempts to model the body physiology of this system, but they have tended to attempt to replicate the system, without building towards a functional understanding of its components. We want to take an approach which is more as if we were engineering a system for a robot, keeping it strictly as simple as possible, and only adding components that might improve its performance. We can use it to answer questions such as why is important that the vasopressin secretion response is linear? Does the ability to control both water and salt excretion improve regulation of osmotic pressure?

A closed system model sufficient to relate the osmotic input signal to the secretory output will require simplified representation of water and salt intake and loss (we will not attempt any distinct model of kidney function or natriuretic mechanisms). Variables will represent intracellular and extracellular water, and salt, with differential equations to define their behaviour. Perturbations will represent drinking and eating. Salt will be lost at a concentration-dependent rate into urine. Water will be lost into urine at a rate dependent on vasopressin, and also at a constant rate, representing nonsensible loss such as respiration. The input to the neurons will be a linear function of osmotic pressure (the ration of body salt to body water), above a set point. Defining the control task in this way will enable us to study how well the system can prolong survival – *i.e.*, when subject to chronic challenge, how long will it maintain osmolarity within tolerable bounds?

## 5 Conclusion

So what about the Forrester flywheel? We know that the vasopressin system is very good at this type of problem. It links release and synthesis to maintain pituitary content (Fitzsimmons et al., 1992), and very efficiently and robustly delivers the right amount of vasopressin, responding to highly variable demand. Our theory is that patterning in the neurons’ spike response to osmostic pressure is an essential element of the system. By building a multiple spiking neuron based model, linked to secretion, we can attempt to investigate this, testing what advantage bursting might give to the dynamics of the secretion response to osmotic input. We hope to demonstrate that a heterogeneous bursting population plays a role in encoding demand, and also in maintaining robust delivery while subject to varying stock levels.

The spiking model described here is able to closely match single cell *in vivo* activity with a fixed input rate. However, to match the *in vivo* response to a changing input with the present model requires that the input affects parameter values. This is avoided by a cousin of the present model described in MacGregor and Leng (2012), which is able to show that asynchronous burst firing does indeed play a role in signal encoding, helping to linearise the response to increasing osmotic input. Current work is attempting to develop a robust secretion model to be integrated with the spiking model. The work will progress to testing a network of model cells, investigating how dendritic communication is used to coordinate their response. Finally, the network will be integrated into a system model of osmotic homeostasis currently under development.

This novel way of understanding the vasopressin system expresses its physiological function in terms of a defined control task, and in so doing it enables systematic, objective study of its performance, and systematic assessment of the utility of each of the various features of the vasopressin system, by evaluating the performance of closely related models in which these specific features are varied systemically. This will generate novel, testable predictions, and subsequent work will test these experimentally. Demonstrating a functional purpose for asynchronous burst firing may apply to other parts of the brain and even apply more broadly to the general problem of distributed control systems.

## Figures and Tables

**Table 1:** Fitted model parameters.

syn_rate_	syn_mag_	*λ*_syn_	*k*_*H*_	*λ*_*H*_	*k*_*A*_	*λ*_*A*_	*k*_*I*_	*λ*_*I*_	*k*_*D*_	*λ*_*D*_	*D*_cap_	*B*_1_	*V*_th_
1240 (Hz)	1.2 (mV)	5 (ms)	−32 (mV)	7.91 (ms)	−0.08 (mV)	1691 (ms)	0.02	12081 (ms)	3.4 (mV)	925 (ms)	12.6 (mV)	0.757	12 (mV)
